# Nanoproteomic analysis of ischemia-dependent changes in signaling protein phosphorylation in colorectal normal and cancer tissue

**DOI:** 10.1186/s12967-015-0752-1

**Published:** 2016-01-08

**Authors:** Florian T. Unger, Nicole Lange, Jana Krüger, Carolyn Compton, Helen Moore, Lokesh Agrawal, Hartmut Juhl, Kerstin A. David

**Affiliations:** Indivumed GmbH, Falkenried 88, 20251 Hamburg, Germany; Biorepositories and Biospecimen Research Branch,National Cancer Institute, National Institutes of Health, Bethesda, MD USA; Arizona State University, Phoenix, AZ USA

**Keywords:** Clinical biospecimen, Ischemia, NanoPro1000, Isoform phosphorylation, Clinical testing

## Abstract

**Background:**

Clinical diagnostic research relies upon the collection of tissue samples, and for those samples to be representative of the in vivo situation. Tissue collection procedures, including post-operative ischemia, can impact the molecular profile of the tissue at the genetic and proteomic level. Understanding the influence of factors such as ischemia on tissue samples is imperative in order to develop both markers of tissue quality and ultimately accurate diagnostic tests.

**Methods:**

Using NanoPro1000 technology, a rapid and highly sensitive immunoassay platform, the phosphorylation status of clinically relevant cancer-related biomarkers in response to ischemia was quantified in tissue samples from 20 patients with primary colorectal cancer. Tumor tissue and adjacent normal tissue samples were collected and subjected to cold ischemia prior to nanoproteomic analysis of AKT, ERK1/2, MEK1/2, and c-MET. Ischemia-induced relative changes in overall phosphorylation and phosphorylation of individual isoforms were calculated and statistical significance determined. Any differences in baseline levels of phosphorylation between tumor tissue and normal tissue were also analyzed.

**Results:**

Changes in overall phosphorylation of the selected proteins in response to ischemia revealed minor variations in both normal and tumor tissue; however, significant changes were identified in the phosphorylation of individual isoforms. In normal tissue post-operative ischemia, phosphorylation was increased in two AKT isoforms, two ERK1/2 isoforms, and one MEK1/2 isoform and decreased in one MEK1/2 isoform and one c-MET isoform. Following ischemia in tumor tissue, one AKT isoform showed decreased phosphorylation and there was an overall increase in unphosphorylated ERK1/2, whereas an increase in the phosphorylation of two MEK1/2 isoforms was observed. There were no changes in c-MET phosphorylation in tumor tissue.

**Conclusion:**

This study provides insight into the influence of post-operative ischemia on tissue sample biology, which may inform the future development of markers of tissue quality and assist in the development of diagnostic tests.

**Electronic supplementary material:**

The online version of this article (doi:10.1186/s12967-015-0752-1) contains supplementary material, which is available to authorized users.

## Background

Reliable development of diagnostic tools, identification of therapeutic targets, and especially accurate molecular stratification of patients all rely on the availability and appropriate collection of human tissue samples [1, 2]. It is well known that the tissue collection procedure itself can have a significant impact on the analysis of data [3, 4]; therefore, understanding and controlling exogenous factors is crucial to the accurate interpretation of results [2, 3, 5]. Furthermore, whereas scientists are familiar with minimizing and controlling experimental variables, they are often unaware of the molecular variability of tissue samples. Studies have described how post-surgical ischemia influences the molecular composition of tissues at the mRNA level [6–8], as well as at the proteome level [9–11]. These observations indicate that exogenous parameters can influence tissue samples in a way that may not reflect the real-life molecular profile of the tissue in vivo.

The effect of intra-operative ischemia on the molecular composition of post-surgical tissues is largely unknown. Intra-operative samples taken prior to vessel ligation may more closely represent the in vivo situation and may be an appropriate reference for the characterization of post-surgical changes due to “cold” ischemia. Available data, although limited, have demonstrated that protein expression patterns are significantly altered in tissues within 30 min post-resection (post-operative ischemia) [9, 11]. Furthermore, protein phosphorylation and gene expression patterns were found to be affected at the time blood supply to the tissue was interrupted during surgery (i.e., intra-operative ischemia) [12, 13]. A better understanding of the effects of intra- as well as post-operative ischemia on tissues will help distinguish the molecular changes that are due to ischemia from those due to disease processes. An improved description of ischemia-induced changes in tissues may ultimately provide a better understanding of how clinically relevant markers have to be interpreted and how this will affect patient stratification, particularly staging, prognosis, and selection of appropriate targeted treatment [14, 15].

Oncology is a medical specialty that has seen considerable progress in the development of targeted therapies. Monoclonal antibodies and small molecules have been developed that target specific receptor tyrosine kinases and intracellular protein kinases, and many are/have been developed alongside a companion diagnostic. As with any new drug or molecular test, the reliability of a companion diagnostic depends upon the tissue sample data used in its development.

NanoPro1000 technology enables the differential quantification of protein isoforms and their phosphorylation status in a single measurement using very small amounts of protein. Protein can be detected at picogram levels, allowing analysis of post-translational modification in samples where limited protein is available [16] (e.g., samples from small-needle biopsies) and making this a valid technology for the clinical setting. In contrast to the commonly used antibody-based assays (e.g., ELISA or western blotting), NanoPro1000 has the potential to characterize protein phosphorylation of multiple sites using just one pan-antibody. NanoPro1000 combines isoelectric focusing of proteins with sensitive chemiluminescence detection with highly specific antibodies, providing a sensitive and quantitative approach to analyzing protein phosphorylation [17]. The approach is fast, with the ability to analyze dozens of samples with multiple antibodies [18].

The current study investigated changes in protein phosphorylation of cancer-related cell signaling molecules in response to tissue ischemia in colorectal tissue using the NanoPro1000 technology and the Meso Scale Discovery technology. The analyzed targets [members of the AKT and MAP-kinase pathways, as well as the receptor tyrosine kinase, c-MET and EGFR (epidermal growth factor receptor)] have clinical relevance in the context of targeted therapy, and therefore the influence of ischemia on these molecules may have an impact on the clinical situation of patients.

## Methods

### Patients

Patients with primary colorectal cancer (CRC) and tumor(s) larger than 3 cm in diameter were enrolled. Patients gave written informed consent for the use and storage of biospecimen and clinical data (Additional file 1: Table S1). Patients with smaller tumors or who had received chemotherapy or radiation therapy less than 3 weeks before surgery were excluded. The study was conducted at two different sites in Hamburg, Germany, and was approved by the competent ethics committee under the reference number PV3342. Therein the use of human biospecimens for research purpose was approved and the compliance with data protection regulations regarding the anonymization of the samples was ensured.

### Tissue collection before and during surgery

**Fig. 1:**
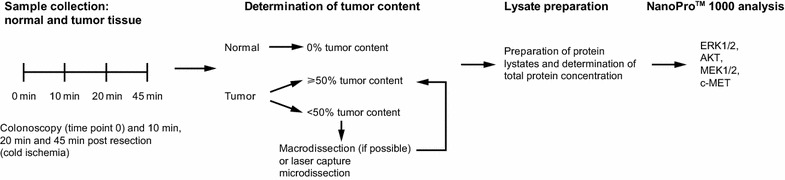
Study workflow indicating cold ischemia time points, quality control of samples, and subsequent proteomic analysis. Patient samples were collected at time point 0 and subjected to cold ischemia. Subsequently, the tumor content of samples was adjusted to ≥50 % and protein lysates were prepared for further proteomic analysis

After induction of anesthesia, patients underwent colonoscopy, during which three biopsies each were taken from the tumor (T) and the adjacent normal tissue (N). (Throughout this manuscript, this time point will be designated as 0). Following resection of the tumor and adjacent normal tissue, 12 tissue samples each were collected from the tumor and the adjacent normal tissue. These 24 tissue samples were divided into three groups and exposed to a cold ischemia time of 10 min (10), 20 min (20), and 45 min (45). Every tissue sample was approximately 5 × 5 × 5 mm and 120 mg. For each of the time points and each tissue type (normal or tumor), half of the samples were immediately stored in the vapor phase of liquid nitrogen, and half were immersion-fixed in 4 % formaldehyde. In this study, only frozen tissue samples were used. Tumor content enrichment to ≥50 % was achieved by either macrodissection or microdissection. Study workflow of sample handling and preparation is shown in Fig. 1.

### Laser capture microdissection (LCM) of colorectal tissues

For this study, LCM was used to isolate and purify cancer cells from any tissue samples with an overall tumor content of <50 %. Any samples not eligible for macrodissection were subjected to LCM. A mean of 15 mm^2^ of LCM material was prepared from each sample to yield sufficient protein for subsequent analyses. The prepared samples were then used for proteomic analysis (see Fig. 1).

### Cell culture of control cells

The human cancer cell lines used as controls in NanoPro1000 experiments were cultured in Dulbecco’s modified Eagle’s medium (DMEM)/Ham’s F12 basal medium (HT29) or RPMI 1640 basal medium (Jurkat) containing 10 % fetal calf serum and 2 mM l-glutamine. Cells were grown in standard conditions at 37 °C and 5 % CO_2_, and signal molecule phosphorylation was stimulated or inhibited by compound treatment. Run controls were included in every run measuring patient material to ensure technical accuracy (Table 1), also see section “Run controls and inter-assay variation”.

**Table 1:** Cell-line run controls

Name	Origin	Treatment	Control	Provider
Jurkat	Human acute lymphocytic leukemia	200 nM PMA for 15 min	Positive for AKT, ERK1/2, and MEK1/2 phosphorylation	Indivumed GmbH
Jurkat	Human acute lymphocytic leukemia	1 µM staurosporine and 50 µM LY294002 for 2.5 h	Negative for AKT, ERK1/2, MEK1/2 phosphorylation	Indivumed GmbH
HT29	Human colon adenocarcinoma	Untreated	Positive for c-MET phosphorylation	Indivumed GmbH
HT29	Human colon adenocarcinoma	Calf intestinal alkaline phosphatase for 50 min	Negative for c-MET phosphorylation	Indivumed GmbH

Compound-treated and compound-untreated cell lines have were as positive and negative controls for protein phosphorylation in order to control run quality. Compound treatment of cell lines is shown

### Preparation of protein lysates from tissues for proteomic analysis

Protein lysates for NanoPro1000 analysis were prepared from fresh frozen tissue blocks. From each sample, 10–15 frozen tissue slices (20 µm) were washed once with cold Dulbecco’s phosphate-buffered saline (DPBS) containing protease-inhibitor cocktails. The protein lysate was prepared by incubating the tissue slices with Tris-lysis buffer, including phosphatase and protease inhibitors, for 30–60 min on ice. To ensure proper mixing during incubation, the tube was vortexed briefly every 10 min, followed by centrifugation at 13,000×*g* for 10 min at 4 °C to remove remaining cell debris. The supernatant was transferred to fresh tubes and stored at −80 °C. An aliquot was used to measure total protein concentration using a bicinchoninic acid (BCA) protein assay.

### NanoPro1000 analysis

The NanoPro1000 system is a new proteomic technology that is used for the detailed characterization of cell signaling processes in extremely small biological samples. It is an automated capillary-based immunoassay platform, which allows for the separation of proteins according to their isoelectric point (pI). Proteins and ampholytes are loaded into the capillary automatically and separated by charge and resolve according to their expected pI values. The separated proteins are then immobilized to the capillary wall via a proprietary, photoactivated capture chemistry. Target proteins are identified using a primary antibody and immunoprobed using an HRP-conjugated secondary antibody and chemiluminescent substrate. The resulting chemiluminescent signal is detected and quantitated. Proteins from complex biological samples are separated by isoelectric focusing to resolve the various modification states of signaling proteins. Analysis of generated data was performed using Compass software v1.8.2 (Cytel, Cambridge, MA, USA). Every NanoPro1000 run includes pI standard ladders in the applied separation gradient for signal calibration. After the completion of a run, standards are set for controls and samples, enabling the determination of the pIs for detected signals. The Compass software then calculates the area under the curve (AUC) and the percentage of the area under the peak (% area) to predefined phosphorylated or unphosphorylated forms of the target. Control lysates and tissue lysates are measured in duplicate with a final protein concentration of 0.15 µg/µl (equal to 60 ng total protein in the capillary). The pan antibodies used (against AKT, ERK1/2, MEK1/2, and c-MET) detect total protein as well as phosphorylated isoforms (Table 2).

**Table 2:** NanoPro1000 assay conditions

Target	Primary antibody, dilution	Secondary antibody	Gradient	Immobilization time (s)
AKT	Anti-AKT (pan), 1:50	Anti-rabbit-HRP	G2 pH 5‒8 (nested)	80
ERK1/2	Anti-ERK 1/2 (pan), ready to use	Anti-rabbit-HRP	G2 pH 5‒8 (nested)	80
MEK1/2	Anti-MEK1/2 (pan), 1:12.5	Anti-mouse-HRP	G2 pH 5‒8 (nested)	80
c-MET	Anti-c-MET (pan), 1:12.5	Anti-rabbit-HRP	G2 pH 5‒8 (nested)	100

Pan antibodies detecting the target signaling molecules were used under optimized assay conditions, as shown. HRP, horseradish peroxidase

*HRP* horseradish peroxidase

### Meso scale discovery analysis

Tissue lysates of freshly frozen specimens from the patients analyzed in this study were prepared by cutting and homogenizing a 20 μm slice from each specimen. Protein concentration of the lysates was determined using a BCA assay (BCA kit; Sigma, Steinheim, Germany). EGFR protein was quantified using 96-well plates with capture antibodies based on the assay platform from Meso Scale Discovery (MSD; Gaithersburg, MD, USA) and the EGFR/pEGFR (Tyr1173) duplex assay kit. Assays were performed using 10 μg of tissue lysate according to the manufacturers’ instructions and analyzed with the SECTOR Imager platform (MSD). Analyses were conducted in triplicate and arithmetic mean values were calculated. Mean values of post-surgery samples were compared with presurgery samples from the same patient. To account for inter-assay variation, lysates of stimulated human cells were used as positive and negative controls.

### Statistical analysis

Protein phosphorylation data were analyzed using Compass software v1.8.2. Data were statistically analyzed using GraphPad Prism 5.0 (GraphPad Software, Inc., La Jolla, CA, USA). Descriptive column statistics of each data set were performed, including the Shapiro–Wilk test for normality. If the Shapiro–Wilk test revealed a non-Gaussian distribution (*p* ≤ 0.05), data sets were tested for significant differences using the Kruskal–Wallis test in combination with the Dunn post-test for multiple comparisons. If the Shapiro–Wilk test revealed a Gaussian distribution (*p* > 0.05), data sets were tested for significant differences using one-way analysis of variance in combination with the Dunnett post-test for multiple comparisons.

## Results

### Patient disposition and clinical data

In a previous part of the study a total of 50 patients with primary CRC who were scheduled for tumor resection surgery consented to study enrollment. A representative cohort of 20 patients was selected for this study, based on high tumor content and overall tissue quality. In total, 20 patients with primary CRC who were scheduled for tumor resection surgery consented to study enrollment. Additional file 1: Table S1 presents patient clinical data including tumor stage and grade. Surgical resection was conducted in two hospitals: the Alten Eichen Hospital, Hamburg, Germany, and the Israelitisches Krankenhaus, Hamburg, Germany. Proteomic analysis of AKT, ERK1/2, MEK1/2, EGFR and c-MET phosphorylation was conducted for normal and tumor ischemic tissue samples from all 20 patients, and relative quantification of phosphorylation was calculated by determining the AUC of annotated peaks in relation to each other (% area).

### Run controls and inter-assay variation

**Fig. 2:**
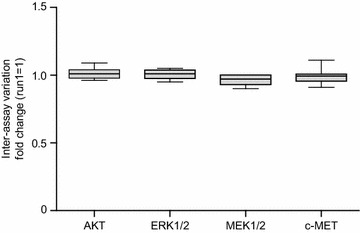
Inter-assay variation of NanoPro1000 assays AKT, ERK1/2, MEK1/2, and c-MET. Data were calculated from run controls of all runs (n = 8). Normalization for individual assays was performed to the first run performed and displayed as *box-and-whisker plots*. *Boxes* represent first and third quartile, *whiskers* represent minima and maxima, and *solid lines within boxes* indicate medians

To ensure the quality of the NanoPro1000 and Meso Scale Discovery assays, run controls were included in every measurement of patient samples. Run controls were then used to evaluate the inter-assay variation of measurements conducted on different days. The positive run controls (high phosphorylation) of the AKT, ERK1/2, MEK1/2, and c-MET assays were each used to calculate the coefficient of variation (CV) of assays for eight individual runs per assay. The inter-assay variation for all assays was low, as shown in Fig. 2. In general, the assays showed a CV below 6 % (AKT, 4.1 %; ERK1/2, 3.5 %; MEK1/2, 4.2 %; and c-MET, 5.7 %).

### Ischemia-dependent regulation of AKT phosphorylation

**Fig. 3:**
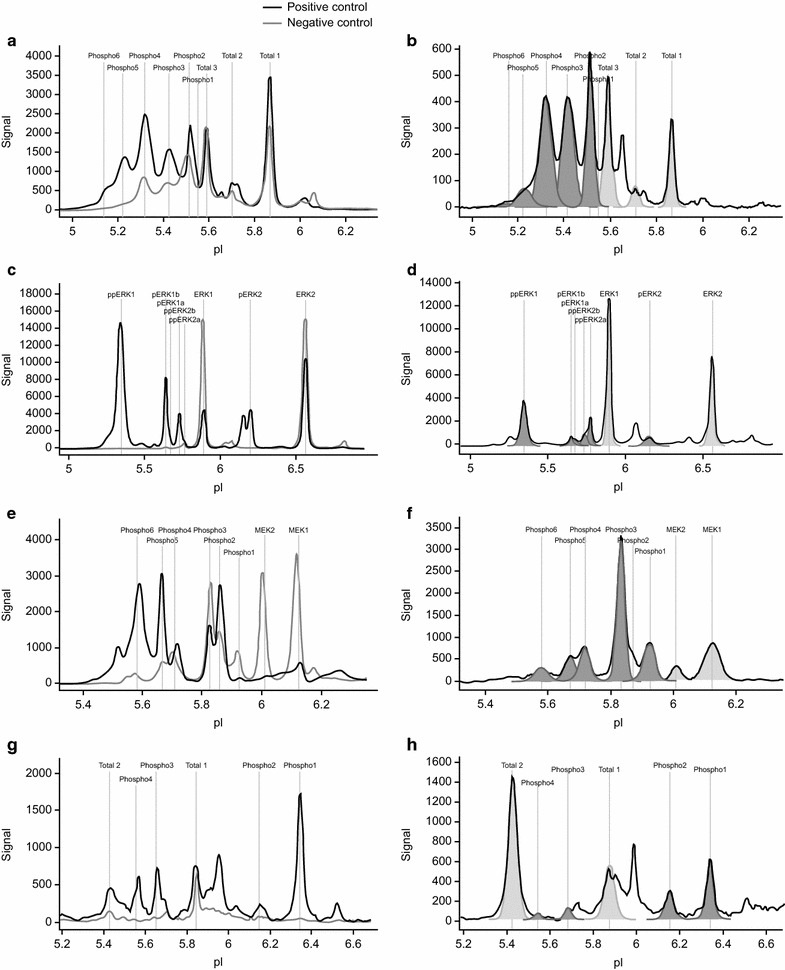
Representative NanoPro1000 chemiluminescence spectra for run controls and patient samples. Spectra overlay of positive (*blue*) and negative (*green*) run controls for AKT, ERK1/2, MEK1/2, and c-MET (**a**, **c**, **e**, and **g**) are shown in order to visualize regulation of phosphorylation. Representative spectra of patient samples (**b**, **d**, **f**, and **h**) are shown, including area under the curve (AUC) for unphosphorylated isoforms (*light gray*) and phosphorylated isoforms (*dark gray*)

**Fig. 4:**
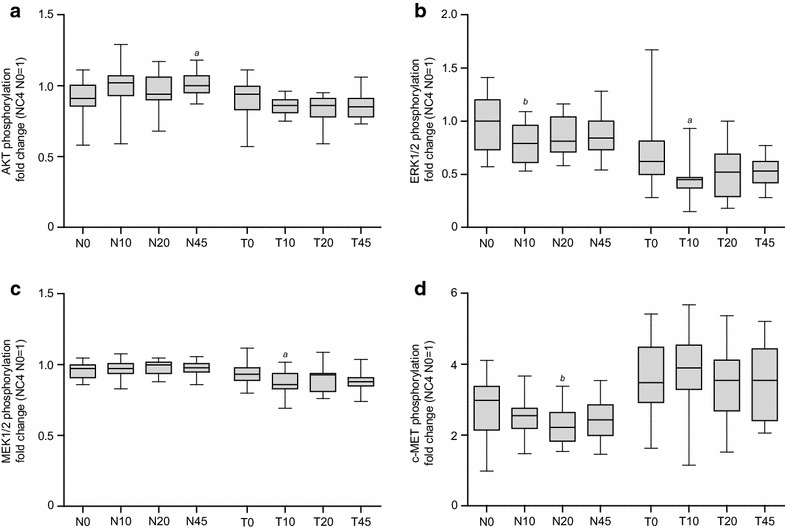
Changes in phosphorylation of AKT, ERK1/2, MEK1/2, and c-MET in response to ischemia. Time-dependent changes in phosphorylation of AKT (**a**), ERK1/2 (**b**), MEK1/2 (**c**), and c-MET (**d**) in normal (N) compared with tumor (T) tissue from 20 patients are shown by *box-and-whisker plots*. *Boxes* represent first and third quartile, *whiskers* represent minima and maxima, *solid lines within boxes* indicate medians. Results were displayed as fold changes normalized in normal tissue to sample NC4/N0 and in tumor tissue to sample NC4/T0. Kruskal–Wallis test and Dunn test for multiple comparisons or analysis of variance (ANOVA) were used for statistical analysis. *N* normal tissue, *T* tumor tissue, *0* before surgery, *10*, *20*, *45* 10, 20, 45 min after resection; ^*a*^ *p* ≤ 0.05; ^*b*^ *p* ≤ 0.01

The phosphorylation of AKT, overall and individual isoforms, was analyzed in relation to ischemia time. The representative AKT spectra and corresponding phosphorylation shown in Fig. 3a, b provide an impression of the generated data from cell-line run controls and patient samples. Therein, unphosphorylated isoforms of AKT are named “total 1–3” and phosphorylated states of these isoforms are named “phospho1-6.” The analysis of overall phosphorylation of AKT in response to ischemia revealed only minor fluctuations in normal and tumor tissue of all patients, as shown in Fig. 4a. In normal tissues, a trend to increased phosphorylation in response to ischemia was detected, which reached statistical significance when comparing N0 and N45.

**Fig. 5:**
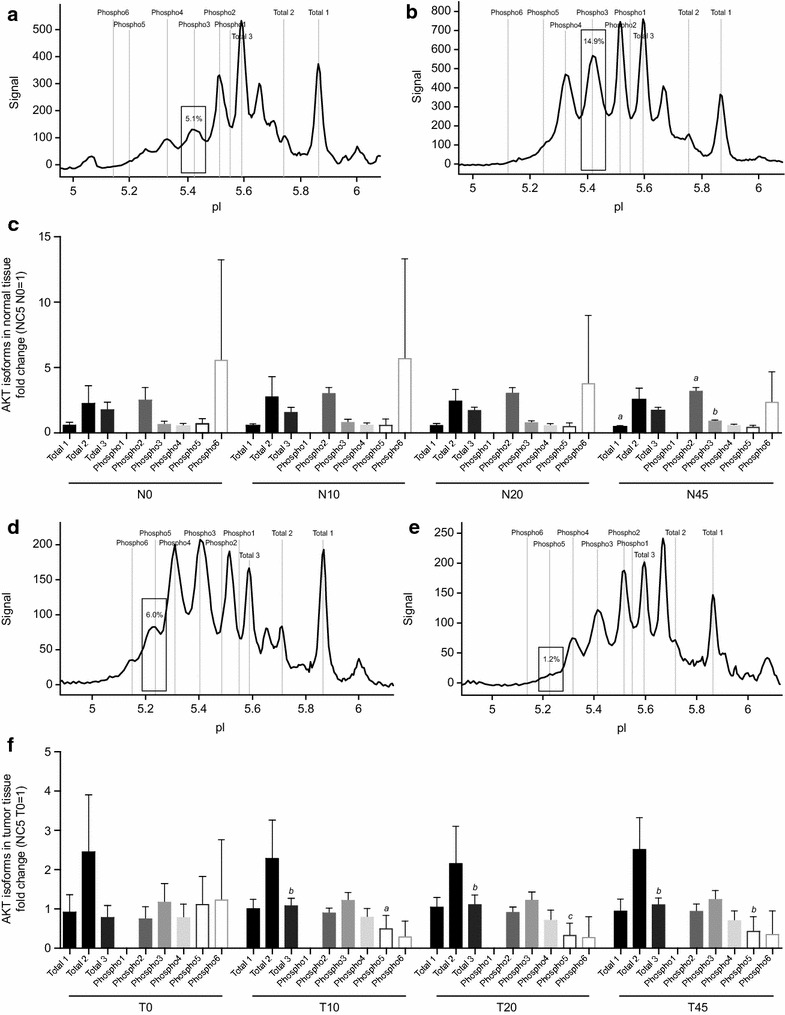
Changes in isoform phosphorylation of the target signaling protein, AKT, in response to ischemia. Representative spectra of time-dependent changes in the phospho3 isoform phosphorylation of AKT between N0 and N45 in normal tissue are shown in **a** and **b**. An overview of all isoforms of AKT in normal tissue of all patients analyzed is shown in **c**; therein results were displayed as fold changes normalized in normal tissue to sample NC5/N0 and in tumor tissue to sample NC5/T0. The same graphs are shown for time-dependent changes between T0 and T20 in the phospho5 isoform in tumor tissue (**d**, **e**). An overview of all isoforms of AKT in tumor tissue of all patients analyzed is shown in **f**. Kruskal–Wallis test and Dunn test for multiple comparisons or analysis of variance (ANOVA) were used for statistical analysis. *N* normal tissue, *T* tumor tissue, *0* before surgery, *10*, *20*, *45* 10, 20, 45 min after resection; Time points (10, 20, 45) are compared with time point 0; ^*a*^ *p* ≤ 0.05; ^*b*^ *p* ≤ 0.01; ^*c*^ *p* ≤ 0.001

In contrast to normal tissue, there was a trend towards decreased AKT phosphorylation in tumor tissue upon cold ischemia (Fig. 4a). Most patient samples (18/19) showed decreased AKT phosphorylation after 20 min of ischemia; however, this trend did not reach statistical significance (data not shown). Regarding the analysis of individual isoforms in relation to ischemia, Fig. 5a, b show representative spectra of changes in isoform phosphorylation between N0 and N45. The analysis of all individual isoforms of AKT in regard to ischemia time revealed statistically significant increased phosphorylation of two isoforms (phospho2 and phospho3), as well as downregulation of one unphosphorylated isoform (total 1) between N0 and N45 in normal tissue (Fig. 5c). Representative spectra for tumor tissue are shown in Fig. 5d, e. Phosphorylation of the isoform, phospho5, was statistically significantly decreased across all ischemia time points compared with T0, whereas phosphorylation of total 3 was significantly increased (Fig. 5f). No statistically significant differences in overall AKT phosphorylation at baseline between normal and tumor tissue were observed.

### Ischemia-dependent regulation of ERK1/2 phosphorylation

Representative ERK1/2 phosphorylation spectra are shown in Fig. 3c, d. Therein, the identity of all peaks in the spectra of the NanoPro1000 ERK1/2 assay are fully elucidated, regarding the state of mono- or dual phosphorylation and assignment to ERK1 and ERK2, respectively. Fluctuations in ERK1/2 phosphorylation detected in normal and tumor tissue samples of all patients in response to ischemia showed more than 0.8-fold changes in single patients (Fig. 4b). The overall phosphorylation of ERK1/2 in individual patients was also determined (data not shown), and an analysis of individual isoforms in relation to ischemia time was performed. The regulation of ERK1/2 phosphorylation was heterogeneous in the analyzed samples with a trend towards decreased phosphorylation in response to ischemia, although single patients showed increased phosphorylation of ERK1/2. In normal tissues, a trend towards decreased phosphorylation in response to ischemia was detected, but only reached statistical significance when comparing time points N0 and N10. The same trend towards decreased ERK1/2 phosphorylation upon cold ischemia was detected in tumor tissue and only showed statistical significance when comparing time points T0 and T10 (Fig. 4b). This trend towards decreased phosphorylation was not statistically significant at other time points.

**Fig. 6:**
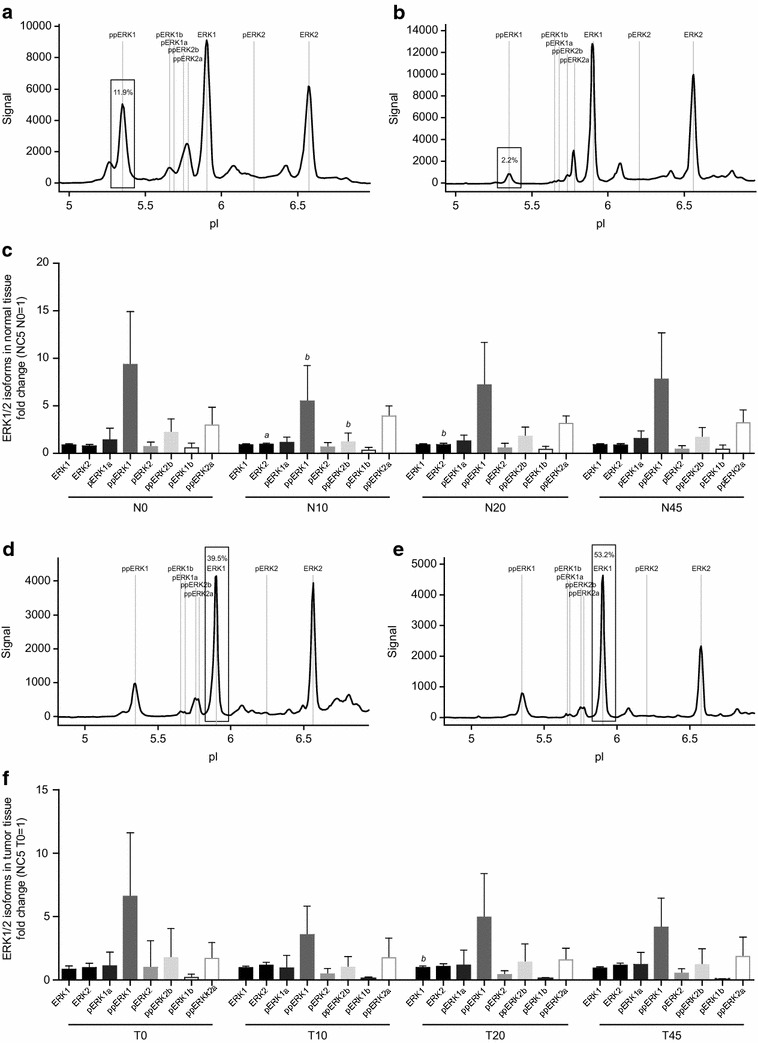
Changes in isoform phosphorylation of the target signaling protein, ERK1/2, in response to ischemia. Representative spectra of time-dependent changes in the ppERK1 isoform phosphorylation of ERK1/2 between N0 and N10 in normal tissue are shown in **a** and **b**. An overview of all isoforms of ERK1/2 in normal tissue of all patients analyzed is shown in **c**; therein results were displayed as fold changes normalized in normal tissue to sample NC5/N0 and in tumor tissue to sample NC5/T0. The same graphs are shown for time-dependent changes in the ERK1 isoform between T0 and T20 in tumor tissue (**d**, **e**). An overview of all isoforms of ERK1/2 in tumor tissue of all patients analyzed is shown in **f**. Kruskal–Wallis test and Dunn test for multiple comparisons or analysis of variance (ANOVA) were used for statistical analysis. *N* normal tissue, *T* tumor tissue, *0* before surgery, *10*, *20*, *45* 10, 20, 45 min after resection; Time points (10, 20, 45) are compared with time point 0; ^*a*^ *p* ≤ 0.01; ^*b*^ *p* ≤ 0.05

Analysis of individual isoforms in regard to ischemia time revealed decreased phosphorylation of two double-phosphorylated isoforms (ppERK1 and ppERK2b) as well as an upregulation of unphosphorylated ERK2 in normal tissue (Fig. 6c). Figure 6a, b show representative chemiluminescence spectra for change in ppERK1 comparing time points N0 and N10. This change in the phosphorylated isoforms reached statistical significance when comparing N0 and N10. Increased unphosphorylated ERK2 was found to be statistically significant when comparing ischemia time points N10 and N20 with N0. In tumor tissue, unphosphorylated ERK1 was found to be statistically significant increased at T20 compared with T0 (Fig. 6f). Representative chemiluminescence spectra of change in ERK1 between T0 and T20 are shown in Fig. 6d, e. There were no other significant changes in ERK1/2 phosphorylation detected in tumor tissue.

**Fig. 7:**
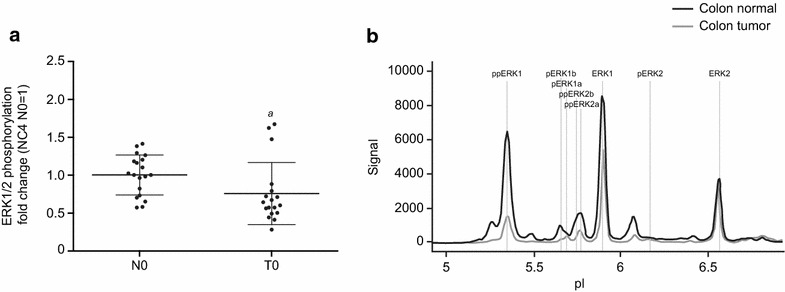
Baseline differences in overall phosphorylation of ERK1/2 in normal versus tumor tissue. The scatter plot (**a**) displays the fold change for individual patients, normalized to NC4 N0, and the mean with standard deviation is indicated. Representative spectra for ERK1/2 in normal and tumor tissue are shown in **b**, indicating the significantly increased abundance of ppERK1 in normal tissue, compared with tumor tissue. Kruskal–Wallis test and Dunn test for multiple comparisons or analysis of variance (ANOVA) were used for statistical analysis. *N* normal tissue, *T* tumor tissue; ^*a*^ *p* ≤ 0.05

At baseline (N and T = 0), a difference in the mean phosphorylation of ERK1/2 was observed between normal tissue and tumor tissue. The mean baseline level of ERK1/2 phosphorylation was significantly (*p* ≤ 0.05) higher in normal tissue (Fig. 7a). The main driver of elevated phosphorylation was the double phosphorylated form of ERK1, ppERK1 (Fig. 7b).

### Ischemia-dependent regulation of MEK1/2 phosphorylation

The phosphorylation of MEK1/2, overall and individual isoforms, was analyzed in relation to ischemia time. Representative spectra of MEK1/2 and corresponding phosphorylation patterns are shown in Fig. 3e, f. Unphosphorylated isoforms of MEK1/2 are named “MEK1 and MEK2,” and phosphorylated states of these isoforms are named “phospho1-6”.

**Fig. 8:**
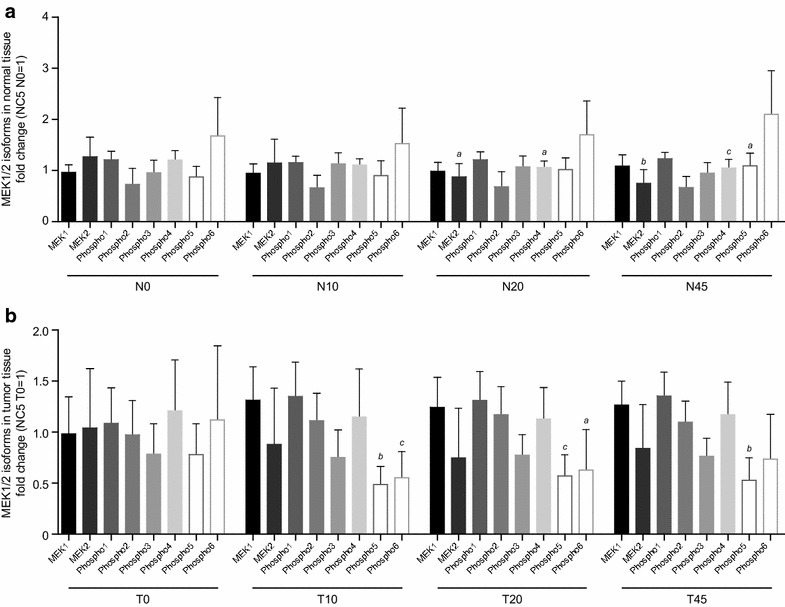
Changes in isoform phosphorylation of the target signaling protein, MEK1/2, in response to ischemia. An overview of ischemia-dependent regulation of isoforms of MEK1/2 in normal tissue of all patients analyzed is shown in **a**. Corresponding data for tumor tissue are shown in **b**. Results were displayed as fold changes normalized to sample NC5/N0 and in tumor tissue to sample NC5/T0. Kruskal–Wallis test and Dunn test for multiple comparisons were used for statistical analysis. *N* normal tissue, *T* tumor tissue, *0* before surgery, *10*, *20*, *45* 10, 20, 45 min after resection; time points (10, 20, 45) are compared to time point 0; ^*a*^ *p* ≤ 0.05; ^*b*^ *p* ≤ 0.01; ^*c*^ *p* ≤ 0.001

Changes in MEK1/2 phosphorylation following ischemia were minimal, with only marginally increased phosphorylation of MEK1/2. In tumor tissue, a trend towards decreased MEK1/2 phosphorylation was detected, and showed statistical significance when comparing time points T0 and T10 (Fig. 4c). This decreased phosphorylation was not statistically significant at other time points. Although there was no statistically significant change in overall phosphorylation of MEK1/2 in normal tissue, some statistically significant changes in the phosphorylation of individual isoforms of MEK1/2 were observed (Fig. 8a). In normal tissue, phosphorylation of the phospho4 isoform decreased in response to ischemia; this reached statistical significance at time points N20 and N45, compared with N0. In contrast, increased phosphorylation of the phospho5 isoform was detected, with statistical significance reached at N45, compared with N0. The unphosphorylated isoform, MEK2, decreased in response to ischemia, reaching statistical significance at N20 and N45, compared with N0.

In tumor tissue, the phospho6 isoform decreased significantly in response to a short period of ischemia of 10 min (T10); this reached statistical significance at T10 and T20, compared with T0 (Fig. 8b). In contrast to normal tissue, decreases in the phospho5 isoforms were statistically significant at all time points (T10, T20, T45), compared with T0. These significant decreases of the phospho5 and phospho6 isoforms contributed to the significant decrease of the overall phosphorylation of MEK1/2 in tumor tissue at the time point T10, compared with T0.

At baseline, no differences were observed in the relative phosphorylation of MEK1/2 in normal tissue compared with tumor tissue. Fluctuations in MEK1/2 phosphorylation due to ischemia time was more heterogeneous in tumor tissue compared to normal tissue, i.e. baseline levels varied more between individual patient samples.

### Ischemia-dependent regulation of c-MET phosphorylation

The phosphorylation of c-MET, overall and individual isoforms, was analyzed in relation to ischemia time. Representative spectra of c-MET and corresponding phosphorylation are shown in Fig. 3g, h. Unphosphorylated isoforms of c-MET are named “total 1” and “total 2,” and phosphorylated states of these isoforms are named “phospho1-4.”

**Fig. 9:**
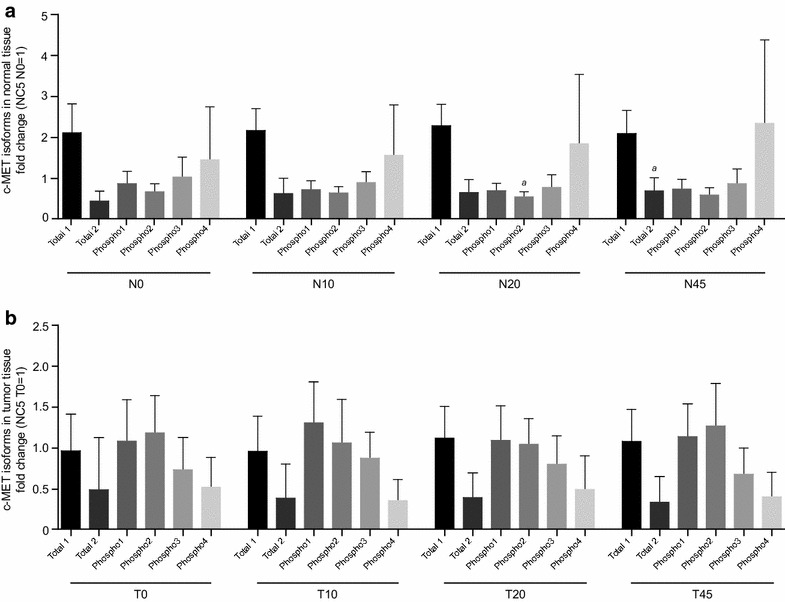
Changes in isoform phosphorylation of the target signaling protein, c-MET, in response to ischemia. An overview of ischemia-dependent regulation of isoforms of c-MET in normal tissue of all patients analyzed is shown in **a**. Corresponding data for tumor tissue are shown in **b**. Results were displayed as fold changes normalized to sample NC5/N0 and in tumor tissue to sample NC5/T0. Kruskal–Wallis test and Dunn test for multiple comparisons were used for statistical analysis. *N* normal tissue, *T* tumor tissue, *0* before surgery, *10*, *20*, *45* 10, 20, 45 min after resection; time points (10, 20, 45) are compared with time point 0; ^*a*^ *p* ≤ 0.05

The results of the NanoPro1000 analysis showed increased fluctuations in c-MET phosphorylation in normal compared with tumor tissue in response to ischemia (Fig. 4d). In normal tissues, a trend towards decreased phosphorylation of c-MET in response to ischemia was detected, reaching statistical significance after 20 min (N20) of ischemia. In tumor tissue, no statistically significant trend towards changes in c-MET phosphorylation upon cold ischemia was detected (Fig. 4d). In normal tissue, significant change in the overall phosphorylation of c-MET was detected following 20 min (N20) of ischemia, which was also present in the analysis of individual isoforms (Fig. 9a). In normal tissue, the phospho2 isoform decreased in response to ischemia; this reached statistical significance at N20, compared with N0. These significant decreases in the phospho2 isoforms contributed to the significant decrease in overall phosphorylation of c-MET in normal tissue at N20, compared with N0. There were no significant changes in the phosphorylation of the c-MET isoform in tumor tissue (Fig. 9b). At baseline, increased phosphorylation of c-MET was observed in normal tissue compared with tumor tissue (Fig. 4d).

### Ischemia-dependent regulation of EGFR phosphorylation

The expression and phosphorylation of EGFR was measured using the MSD technology. There were no significant changes in expression and/or phosphorylation of the receptor in relation to ischemia time (Additional file 2: Figure S1), for both normal and tumor tissue samples from the 20 patients analyzed in this study.

## Discussion

The identification of biomarkers for the stratification of cancer patients by stage of disease, prognosis, and administration of the most appropriate targeted anticancer treatment depends upon the availability and collection of human biospecimens. Biospecimens used for patient stratification in the clinical setting have to reflect the molecular reality in cancer patients and not be biased by factors such as ischemia. Anticancer treatments targeting specific molecular markers are widely used for the clinical management of patients with CRC. One of these molecular targets is the EGFR and its downstream signaling molecules within the AKT and MAPK pathways [19]. In addition to the mutation status of EGFR itself, and downstream signaling molecules like KRAS, the proteomic analysis of kinase activation becomes more and more clinically relevant. Compared with the linear output of mutation analysis, the multidimensional information obtained by proteomic pathways analysis may be useful when analyzing reasons for therapeutic resistance and sample heterogeneity, even in patient cohorts with similar genotype [20–22].

In the current study, normal and tumor tissue from 20 selected patients with CRC were analyzed for ischemia-induced changes in protein phosphorylation and for changes in the phosphorylation of isoforms of selected proteins. The analyzed proteins, ERK1/2, AKT, MEK1/2, EGFR and c-MET are potentially clinically relevant molecular markers for the characterization of tumors according to prognosis [23–25] and anticancer therapy [26, 27]. The analysis was conducted using the relatively novel antibody-based NanoPro1000 technology. This technology enables the detection of overall phosphorylation of proteins as well as the phosphorylation of individual isoforms. Since isoforms of signaling proteins have distinct biological functions [28–30], it is even more important to analyze ischemia-induced changes and thus identify markers or signatures indicating alteration of molecular targets. In order to detect tumor-specific signals, the tumor content of samples used in this study was adjusted to ≥50 % by macrodissection or microdissection, which avoids strong dilution of tumor-specific signals by normal cells and tissues present in the sample. This was shown to be particularly important in this study given that the baseline level of ERK1/2 phosphorylation was higher in normal tissue compared with tumor tissue. In this study, inter-assay variation, as measured using run controls, was below 10 percent. This indicates that the assays were robust and reproducible in the time frame of the study and that small changes in protein phosphorylation were detectable, even when comparing runs on different days.

In this study, ischemia-induced changes in overall phosphorylation of the signaling proteins analyzed were not as large as expected. However, we were able to identify significant changes in isoform phosphorylation that can be potentially assigned to specific phosphosites and later be interpreted in a biological context. Furthermore, we showed different degrees of baseline protein phosphorylation when comparing normal and tumor tissue, indicating the importance of analyzing tumor content of tissue samples. Specifically, the phosphorylation of c-MET, a heterodimeric transmembrane receptor [31], has in this study been found to be significantly decreased after 20 min of ischemia in normal tissue, whereas no change has been detected in tumor tissues. The differences in phosphorylation between tumor tissue and normal tissue might be explained by a higher and more heterogeneous baseline level of phosphorylation of c-MET in tumor tissue. This heterogeneity might reflect patients` individuality as well as molecular changes due to cancer, which may disguise any significant change due to ischemia in the whole cohort of tumor patients. The analysis of isoforms of c-Met revealed statistically significant changes in phospho2 following 20 min of ischemia in normal tissues (see Fig. 9), which is in accordance with the regulation of the overall phosphorylation level. It is unclear whether patients with high baseline levels of c-MET phosphorylation respond to ischemia differently than patients with no c-MET phosphorylation at baseline. In addition, the presence of high levels of c-MET protein and RNA expression does not correlate with *MET* gene amplification or pathway activation [32].

The EGFR (ErbB-1) is a member of the ErbB family of receptors, and a clinically targeted receptor tyrosine kinase. Although the expression and phosphorylation of this receptor has not been found to be influenced by ischemia, downstream signaling of the receptor via the Akt and/or MAPK pathways was influenced by ischemia. For example AKT, the key signaling protein of the AKT pathway, displayed significantly increased levels of phospho2 and phospho3 isoform phosphorylation in normal tissue and decreased levels of phospho5 phosphorylation in tumor tissue. Levels of individual AKT isoforms have been shown to correlate with patient prognosis and response to anticancer therapy [33, 34]. Changes in isoforms may be the main driver of changes in overall phosphorylation. In this study, changes in isoform phosphorylation reached significance at some time points, whereas changes in overall phosphorylation did not. Since AKT inhibits apoptosis and regulates cell growth, survival, and proliferation, and facilitates recovery from ischemia, one could have expected increased levels of AKT phosphorylation in response to ischemia, as seen in normal tissues.

Crosstalk exists between the PI3K-AKT pathway and the MAP-kinase pathway. These are signal transduction pathways that involve a chain of dual-specificity kinases activating each other in series. MAP-kinase pathway dysregulation is common during oncogenesis, as well as in other diseases [35]. In this study, MEK1/2 phosphorylation was found to be downregulated in tumor tissue only after 10 min of ischemia, whereas no statistically significant change in phosphorylation was detected in normal tissue. Although there was no significant change in the overall phosphorylation of MEK1/2 in normal tissue in response to ischemia, the analysis of phosphorylation levels of individual isoforms revealed a statistically significant decrease in the MEK1/2 isoform phospho4 and an increase in phospho5. This inverse relationship between the two isoforms could be explained by the assumption that the regulated phosphosites could either be inhibiting or activating protein activity. In tumor tissue, two MEK1/2 isoforms have been found to be significantly downregulated, namely phospho5 and phospho6. In general, the heterogeneity of MEK1/2 phosphorylation levels in tumor as well as normal tissue at baseline was low compared with the other signaling proteins analyzed, indicating a low heterogeneity between individual patients.

Results also revealed decreased phosphorylation of ERK1/2 in response to ischemia in normal as well as tumor tissue after 10 min of ischemia, similar to MEK1/2 in tumor tissue. The decreased phosphorylation in normal tissue after 10 min of ischemia was greater than in tumor tissue and accompanied by statistically significant changes in the phosphorylation of the ppERK2b and ppERK1 isoforms (see Fig. 7a, b).

In addition, the detected differences in baseline phosphorylation levels of ERK1/2 when comparing normal and tumor tissue is in contrast to the often assumed hyperactivation of the MAP-kinase pathway in cancer. Consequently, this is of particular importance for the analysis of clinical biospecimens, since they comprise a mixture of normal and tumor cell populations as well as stromal tissue and immune cells. Thus, contamination of tumor-specific ERK1/2 will occur when analyzing biospecimens with unknown tumor content, potentially leading to false results, with consequences for the clinical management of disease.

## Conclusion

The data generated in this study differ compared with data described in a previous study using other technologies [9]. In general, the comparison of protein phosphorylation data from different studies, using different technologies, is hindered by the specificity of antibodies and the molecular technique used. For example, the immobilization of proteins within different molecular techniques (e.g., immunohistochemistry, western blot, ELISA) varies considerably. Different protein modifications in turn may influence the binding of the antibody to its target protein. The possibility to check the antibody for specificity prior to conducting experiments is critical in order to generate valid results. Furthermore, the NanoPro1000 technology uses pan antibodies directed against target proteins to detect unphosphorylated and phosphorylated forms of the protein simultaneously, circumventing the use of different antibodies (total and phospho-specific antibodies). In future studies, the regulation of individual isoforms of signaling proteins has to be elucidated in terms of the identity of regulated phosphosites (e.g. Ser473 for Akt), in order to be able to assess the biological impact of the regulation (i.e., activating or inhibiting kinase activity) and impact on anticancer therapy. This will give further insights into the effects of ischemia on tissues, and will determine the types of phosphorylation of signaling molecules that are activated by cells themselves, and the types that occur when cells die due to ischemia. This will be achieved by further experiments using isoform- and phospho-specific antibodies, as well as phosphatase treatments.

In conclusion, this study provides new insights into the biology of ischemia-induced changes in normal and tumor tissue. This may have clinical relevance in terms of identifying markers of tissue quality, stratification of patients and other relevant parameters.

## Additional files

10.1186/s12967-015-0752-1 Clinical data for the 20 patients analyzed in the study. Presents patient clinical data including tumor stage and grade.
10.1186/s12967-015-0752-1 Unaltered expression and/or phosphorylation of the target signaling protein, EGFR, in response to ischemia. An overview of ischemia-dependent regulation of EGFR in normal and tumor tissue of all patients, analyzed by the MSD technology, is shown. ANOVA, Kruskal–Wallis test and Dunn test for multiple comparisons were used for statistical analysis. There were no significant changes detected.

## Abbreviations

AUC: area under the curve
BCA: bicinchoninic acid
CRC: colorectal cancer
CV: coefficient of variation
DMEM: Dulbecco’s modified Eagle’s medium
DPBS: Dulbecco’s phosphate-buffered saline
EGFR: epidermal growth factor receptor
LCM: laser capture microdissection
