# Supplementary material for: Nanoproteomic analysis of ischemia-dependent changes in signaling protein phosphorylation in colorectal normal and cancer tissue
Source: J Transl Med. 2016 Jan 8;14:6. doi: 10.1186/s12967-015-0752-1 (PMC4705760; doi:10.1186/s12967-015-0752-1)
Supplement: Supplementary file 1 — 10.1186/s12967-015-0752-1 Clinical data for the 20 patients analyzed in the study. Presents patient clinical data including tumor stage and grade. [file 12967_2015_752_MOESM1_ESM.docx]

Additional table 1. Clinical data of patients.

a = Alten Eichen-Hospital Hamburg; b = Israelite Hospital Hamburg; f = female; m = male

| **Case no.** | **Age [yrs]** | **Gender** | **Hospital** | **Description** | **Tumor size [cm]** | **Histological type** | **Grading** | **Stage** |
| --- | --- | --- | --- | --- | --- | --- | --- | --- |
| **NC4** | 54 | f | a | Malignant neoplasm of sigmoid colon | 1.8 | Moderately differentiated adenocarcinoma | G2 | I |
| **NC5** | 71 | m | a | Malignant neoplasm of rectum | 7 | Moderately differentiated carcinoma | G2 | IVA |
| **NC11** | 62 | m | a | Malignant neoplasm of sigmoid colon | 7 | Poorly differentiated adenocarcinoma | G3 | IIA |
| **NC12** | 66 | f | a | Malignant neoplasm of rectum | 4.5 | Poorly differentiated adenocarcinoma | G3 | IVA |
| **NC16** | 69 | f | a | Carcinoma in situ of rectum | 7 | Moderately differentiated adenocarcinoma | G2 | 0 |
| **NC21** | 69 | m | b | Malignant neoplasm of sigmoid colon | 4,5 | Moderately differentiated adenocarcinoma | G2 | IIA |
| **NC25** | 45 | m | b | Malignant neoplasm of rectum | 6 | Moderately differentiated adenocarcinoma | G2 | IIIB |
| **NC26** | 71 | f | b | Malignant neoplasm of sigmoid colon | 9 | Poorly differentiated adenocarcinoma | G3 | IVB |
| **NC27** | 64 | m | b | Malignant neoplasm of sigmoid colon | 4.5 | Moderately differentiated adenocarcinoma | G2 | n/a |
| **NC30** | 71 | f | b | Malignant neoplasm of sigmoid colon | 7 | Moderately differentiated adenocarcinoma | G2 | I |
| **NC35** | 74 | f | b | Malignant neoplasm of descending colon | 3 | Moderately differentiated, partially mucinous adenocarcinoma | G2 | IIIA |
| **NC36** | 61 | f | b | Malignant neoplasm of sigmoid colon | 4.5 | Moderately differentiated adenocarcinoma | G2 | I |
| **NC37** | 65 | f | b | Malignant neoplasm of rectum | 5 | Poorly differentiated adenocarcinoma | G3 | I |
| **NC39** | 70 | m | b | Malignant neoplasm of rectum | 6 | Moderately differentiated mucinous adenocarcinoma | G2 | IIA |
| **NC40** | 66 | f | b | Malignant neoplasm of rectum | 6 | Moderately differentiated adenocarcinoma | G2 | I |
| **NC41** | 88 | m | b | Malignant neoplasm of rectum | 3.5 | Moderately differentiated adenocarcinoma | G2 | IVA |
| **NC42** | 76 | m | b | Malignant neoplasm of sigmoid colon | 3 | Poorly differentiated adenocarcinoma | G3 | III B |
| **NC43** | 72 | f | b | Malignant neoplasm of sigmoid colon | 3.5 | Moderately differentiated adenocarcinoma | G2 | IIA |
| **NC47** | 83 | f | b | Malignant neoplasm of sigmoid colon | 4 | Moderately differentiated adenocarcinoma | G2 | I |
| **NC48** | 74 | m | b | Malignant neoplasm of sigmoid colon | 4.5 | poorly differentiated mucinous adenocarcinoma | G3 | IIA |
