# Supplementary material for: Nanoproteomic analysis of ischemia-dependent changes in signaling protein phosphorylation in colorectal normal and cancer tissue
Source: J Transl Med. 2016 Jan 8;14:6. doi: 10.1186/s12967-015-0752-1 (PMC4705760; doi:10.1186/s12967-015-0752-1)
Supplement: Supplementary file 2 — 10.1186/s12967-015-0752-1 Unaltered expression and/or phosphorylation of the target signaling protein, EGFR, in response to ischemia. An overview of ischemia-dependent regulation of EGFR in normal and tumor tissue of all patients, analyzed by the MSD technology, is shown. ANOVA, Kruskal–Wallis test and Dunn test for multiple comparisons were used for statistical analysis. There were no significant changes detected. [file 12967_2015_752_MOESM2_ESM.pdf]

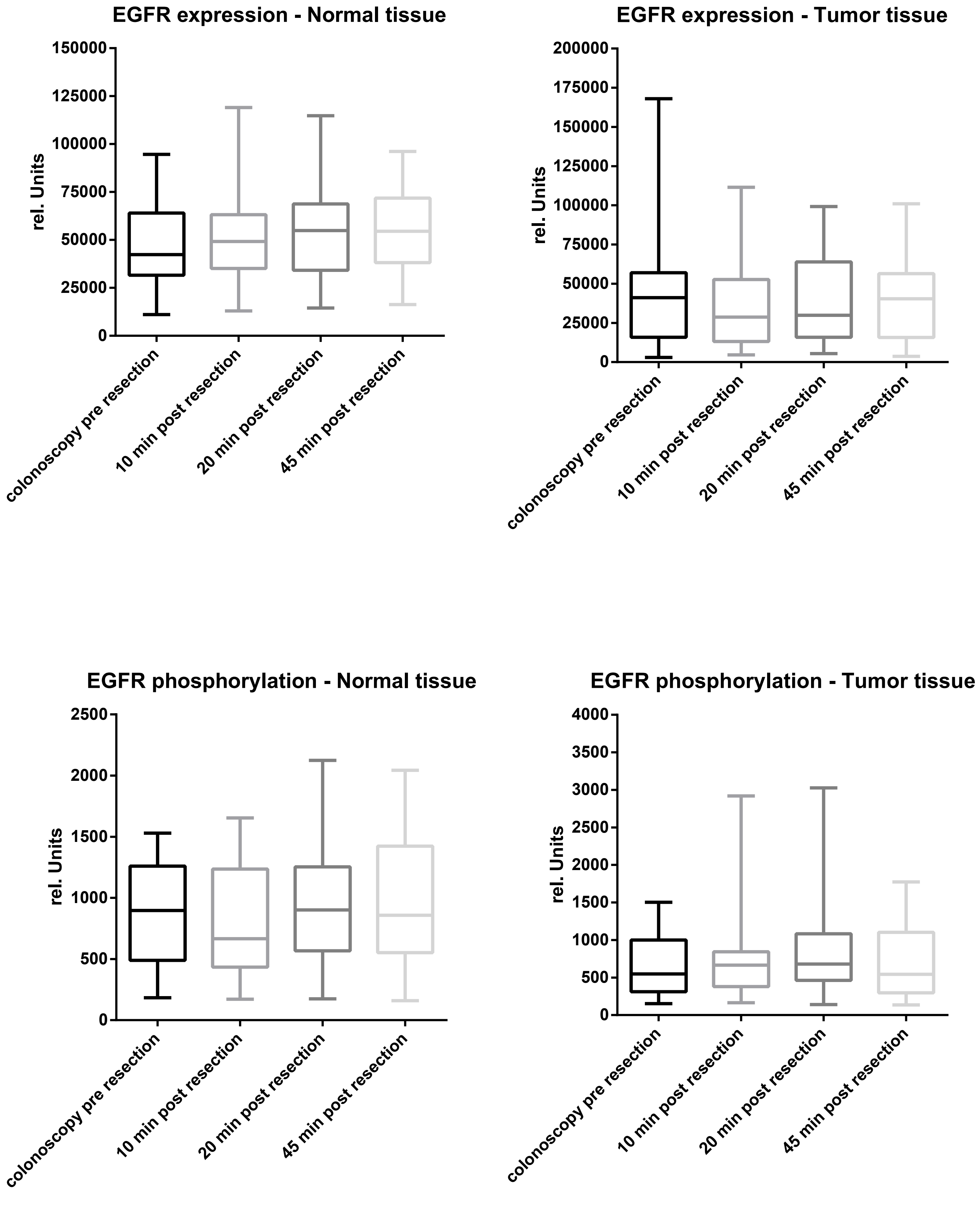

Additional figure: Unaltered expression and/or phosphorylation of the target signaling protein, EGFR, in response to ischemia. An overview of ischemia-dependent regulation of EGFR in normal and tumor tissue of all patients, analyzed by the MSD technology, is shown. ANOVA, Kruskal-Wallis test and Dunn test for multiple comparisons were used for statistical analysis. There were no significant changes detected.
